# Authenticity is more than self-enhancement: behavioural and neurophysiological evidence

**DOI:** 10.1093/scan/nsaf103

**Published:** 2025-10-14

**Authors:** Chengli Huang, Emily K Penney, Constantine Sedikides, Nicholas J Kelley

**Affiliations:** School of Mental Health, Wenzhou Medical University, Wenzhou, 325035, China; Centre for Research on Self and Identity, University of Southampton, Southampton, SO17 1BJ, United Kingdom; Centre for Research on Self and Identity, University of Southampton, Southampton, SO17 1BJ, United Kingdom; Centre for Research on Self and Identity, University of Southampton, Southampton, SO17 1BJ, United Kingdom; Centre for Research on Self and Identity, University of Southampton, Southampton, SO17 1BJ, United Kingdom

**Keywords:** authentic self, presented self, self-enhancement, self-consistency, self-accuracy, emotional Stroop effect

## Abstract

Negative self-descriptive information can be threatening to the self. This may depend, however, on the self-representation for which the information is relevant. We focused on two self-presentations, the authentic self and the presented self. In particular, we examined how the authentic and presented selves are influenced by emotional self-descriptiveness. Participants (*N *= 147) completed a self-referent emotional Stroop task while electroencephalogram (EEG) was recorded. They viewed in coloured text positive or negative traits exemplifying the authentic self (‘I am genuinely honest’), the presented self (‘I am outwardly honest’), or control (‘It is clearly honest’). Colour naming latency was slower to negative (vs. positive) traits for the presented self and control. Colour naming latency was faster to negative (vs. positive) traits for the authentic self. Event-related potentials indicated that at both early (P1) and later (P3) stages of attentional processing, the authentic self exhibited comparable amplitudes to negative and positive traits. However, P1 was larger for negative, and P3 was larger for positive, traits for the presented self. Taken together, the findings highlight that the presented self is more pursuant of positivity, whereas the authentic self is more tolerant of negativity.

The self-concept is multifaceted (Marsh 1990, Sande 1990). A key facet involves the internal-external distinction. William James (1890) pioneered this distinction in terms of the spiritual (internal) and material or social (external) self. Contemporary theorists have framed the distinction as one between the private and public self (Baumeister 1986, Fenigstein 2009). Individuals are motivated to perceive the external expressions of the self (henceforth ‘the presented self’) in a positive light, extolling their strengths and underemphasizing their weaknesses (Baumeister 1982, Leary 1995). Whereas some researchers suggest that self-enhancement motivation extends to aspects of the internal self (henceforth ‘the authentic self’), others propose that the authentic self is driven by self-consistency or self-accuracy motivation (Sedikides and Schlegel 2024). We test these theoretical views by examining how negative (vs. positive) self-relevant information captures attention, both behaviourally and neurophysiologically, when the presented and authentic selves are salient.

## The authentic self

Authenticity is ‘the subjective perception that one is being the true, unvarnished “me”’ (Sedikides et al. 2019, p. 73). Despite the construct’s long history, dating back to Aristotelian thinking (Tredennick and Thomson 1976), its meaning has been a matter of controversy (Baumeister 2019, Hicks et al. 2019). A traditional view conceptualized authenticity through the lens of *self-accuracy*, the motivation to form an accurate image of the self or unbiasedly process self-relevant information (Kernis and Goldman 2006). In support of this view, individuals high on authenticity are less defensive when faced with evidence that their behaviour is short of their ideals (Lakey et al. 2008). However, self-accuracy is difficult to attain or measure (Vazire and Wilson 2012). Moreover, the more unbiased individuals believe themselves to be, the more likely they are to report possessing more positive than negative characteristics, casting doubt on the veracity of their self-beliefs (Gillath et al. 2010). Another view conceptualizes authenticity through the lens of *self-consistency*, the motivation to maintain coherence among one’s cognitions, emotions, and behaviours (Kernis and Goldman 2006, Wood et al. 2008). In support of this view, inconsistency between one’s gender identity (female) and experimentally allocated self-presentation (masculine) reduces authenticity (Dormanen et al. 2020). However, individuals appraise their socially desirable behaviours as authentic, whether or not they align with their self-concept (Sheldon et al. 1997), and consider their positive behaviours as more authentic than negative ones (Jongman-Sereno and Leary 2016). These findings challenge the self-consistency view but are readily explained by the self-enhancement view.

According to the self-enhancement view, the authentic self encompasses predominantly positive characteristics. Consequently, individuals process self-relevant information so as to accentuate their strengths and downplay their weaknesses (Guenther and Sedikides, in press). In support of this view, individuals evaluate their true self as moral and positive (Strohminger et al. 2017), and label the times when they behaved in accordance with a positive (vs. negative) trait as authentic (Bailey and Iyengar 2023). Similarly, the more positively individuals assess a change in their lives, the more likely they are to think that this change was fuelled by authenticity (Bench et al. 2015). Finally, laboratory experiments, individual difference studies, and daily diary studies point to reciprocal positive associations between authenticity and self-enhancement (Guenther et al. 2024).

## The presented self

The presented self is a mental representation as integral to one’s self-concept as the authentic self. Stakes are high for the presented self, given that it can facilitate or undermine cooperation, reputation, respect, status, as well as access to social networks, professional opportunities (e.g. jobs, promotions, and housing), and personal resources (e.g. friends and partners; Vonasch et al. 2018, Dores Cruz et al. 2021). Hence, self-presentation promotes a sanitized portrait of the individual, exaggerating, if not glorifying strengths, while minimizing, if not concealing, weaknesses (Baumeister 1982, Hancock and Toma 2009).

Humanistic theories and person-centred therapies propose that individuals often modify or distort their social behaviour to conform to perceived ‘conditions of worth’ imposed by their surrounding environment (Rogers 1951, Tunnel 1984). For instance, smiling at an unfunny joke to fit in the social gathering, or feigning enthusiasm for the company’s mission during a job interview, might be motivated by the desire to evade negative evaluations from others. Empirical research supports this notion, as individuals often engage in strategic self-presentation, denying negative traits and drawing attention to positive ones (Roth et al. 1986, Lee et al. 1999). Furthermore, positive self-relevant contexts can elicit a smaller N400 than positive other-relevant contexts, suggesting that individuals are more likely to expect positive information when referring to themselves (Fields and Kuperberg 2015).

## Current investigation

### Emotional Stroop task

**Figure 1.**
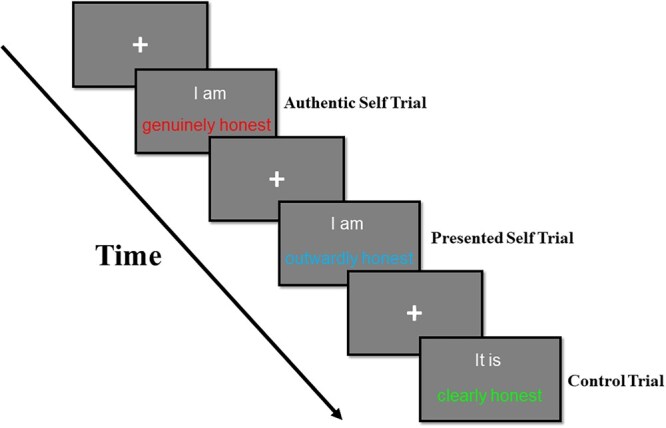
Trial event diagram.

To test the three views of authenticity, we recorded behavioural and neurophysiological (event-related potential or ERP) responses to emotionally charged self-evaluations in a modified Stroop task, the Emotional Stroop Task (Mathews and MacLeod 1985, Watts et al. 1986). Here, participants identify the ink colour of emotionally evocative (or neutral) words (Fig. 1). Both the traditional Stroop and Emotional Stroop tasks demonstrate that the meaning of the text captures attention, even when it is irrelevant to the task. The classic Stroop effect arises from a direct semantic conflict (or incongruence) between word meaning and ink colour (e.g. the word ‘red’ printed in green vs. red ink). In contrast, the emotional Stroop effect delves into the discrepancy between emotionally salient versus neutral words (e.g. ‘death’ vs. ‘door’ printed in red), without semantic conflict central to the classic Stroop effect. This fundamental distinction positions the emotional Stroop task as a unique paradigm: where the traditional Stroop task reveals competition between automatic reading and controlled colour-naming processes, the emotional Stroop task reflects a generalized slowing due to threat detection mechanisms (Algom et al. 2004). Consequently, the emotional Stroop effect refers to the slowdown in responding to the ink colour of negative (vs. positive or neutral) words (Williams et al. 1996, Bar-Haim et al. 2007, Phaf and Kan 2007), resulting from the automatic attention allocation to threatening stimuli at the expense of concurrent task demands (Öhman 1993, Öhman et al. 2001). Moreover, such negativity occurs rapidly and automatically in emotion information processing (Pratto and John 1991, Anderson et al. 2003, Zald 2003) and is stronger than positivity (Baumeister et al. 2001). For example, the motivation to avoid negative self-definitions typically outweighs the drive to pursue positive self-definitions (Sedikides 2012, Sedikides et al. 2016). Leveraging the emotional Stroop effect’s sensitivity to negativity, we used this task to assess the resistance of the authentic and presented self to negativity.

The presented self is predominantly shaped by self-enhancement motivation, which promotes linking the self-concept to positive than negative information (Baumeister 1982, Leary and Kowalski 1990, Sedikides and Gregg 2008). Hence, negative self-relevant information will disproportionately affect self-representation. Specifically, when maintaining a positively biased self-presentation, exposure to negative self-descriptors creates greater cognitive-affective dissonance than in the authentic self, thereby amplifying their salience and disruptive potential. As such, we hypothesized that in an emotional Stroop task, interference of negative self-evaluations would be more pronounced for the presented self (an amplified emotional Stroop effect). The case for the authentic self is equivocal. On the one hand, building on the self-consistency/self-accuracy views, individuals would integrate rather than avoid negative self-aspects, and such balanced processing of all self-relevant information would decrease their attentional salience to negativity and thus minimize defensive responses to negative self-representation. If so, the interference of negative self-evaluations would be less pronounced for the authentic than presented self (an attenuated emotional Stroop effect relative to the presented self, but amplified compared to the control condition). On the other hand, self-enhancement motivation might drive the authentic self, much like the presented self. If so, the interference of negative self-evaluations would be on par with the effect observed for the presented self (an amplified emotional Stroop effect).

### Event-related potential assessment

We also recorded electroencephalography (EEG) activity during the emotional Stroop task. Prior ERP research has delineated distinct temporal stages of emotional information processing: the P1, which reflects initial threat detection; the N170 and early posterior negativity, which reflect emotional and non-emotional discrimination; and the later components (the P3 and late positive component), which distinguish between positive and negative words (Luo et al. 2010, Zhang et al. 2014). This temporal framework provides a neurocognitive basis for examining emotional processing dynamics in our self-referential Emotional Stroop task. Taken together, we focused on the ERPs at early-stage selective attention (P1; Batty and Taylor 2003, Pourtois et al. 2005, Zhang et al. 2014), attention allocation to emotionally evocative stimuli (N170; Williams et al. 2006, Zhang et al. 2014, Cai et al. 2016), and early (P2; Fields and Kuperberg 2012, Fan et al. 2016, San Martín et al. 2016) as well as late (P3; Gray et al. 2004, Tacikowski and Nowicka 2010) stages of self-relevant information processing. We describe the pertinent components below.

*P1*. The P1 is an early sensory-evoked component, emanating from parieto-occipital regions as early as 60 ms post-stimulus (Luck 2014). It reflects the selective amplification of sensory information (Hillyard et al. 1998) and is sensitive to emotional stimuli (Mueller et al. 2013, Schindler and Bublatzky 2020), with larger P1 amplitudes evoked by negative compared to neutral stimuli (e.g. faces, words; Batty and Taylor 2003, Van Hooff et al. 2008, Luo et al. 2010) indicating that the P1 can differentiate between threatening and non-threatening information (Zhang et al. 2014, Huang et al. 2025). The P1 emerges during the initial phase, representing an evolutionary adaptation for threat vigilance (Luo et al. 2010, Zhang et al. 2014). Such early emotional response might signify rapid extraction of emotion-related information and function, at least in part, independently of subsequent emotional processes (N170; Vuilleumier and Pourtois 2007).

*N170*. The N170 is a negative deflection that typically peaks approximately 170 ms after stimulus onset over lateral occipito-temporal regions (Luck 2014). The N170 reflects early rapid attention to visual stimuli (Rossion and Jacques 2012), with larger N170 amplitudes representing the allocation of more attentional resources (Cai et al. 2016). Moreover, the N170 is modulated by emotional stimuli, with a substantially enhanced amplitude for emotional relative to neutral stimuli (Williams et al. 2006, Luo et al. 2010, Zhang et al. 2014, Schindler et al. 2023), especially for negative (vs. positive) ones (Montalan et al. 2008, Cai et al. 2016).

*P2* **.** The P2 is a positive deflection spanning 150–250 ms over the anterior-central region (Luck 2014). It exhibits greater amplitudes in response to stimuli containing target features, indicating early selective attention towards task-relevant stimuli (Potts 2004, Potts et al. 2006). This amplification is pronounced when the targets are infrequent (Luck and Hillyard 1994, Glazer and Nusslock 2022). Furthermore, the P2 has been associated with emotional processing, suggesting that its role in modulating selective attention is influenced by emotional content (Kotz and Paulmann 2011, Hajcak et al. 2012). However, findings regarding the modulation of P2 by emotion are mixed. Although some studies reported increased P2 amplitudes with emotional stimuli compared to neutral ones, others found the opposite pattern (Schindler and Bublatzky 2020). Unlike the earlier emotion-detection stages represented by the P1 and N170, the P2 is associated with higher-order, deeper, and conscious emotional processing (Prete et al. 2015, 2018, Nie et al. 2020). Moreover, research has implicated the P2 in self-referential processing, but findings are inconsistent. Some studies obtained reduced P2 amplitudes for self-related stimuli (Keyes et al. 2010, Liu et al. 2019), whereas others reported increased amplitudes (Fields and Kuperberg 2012, Fan et al. 2016, San Martín et al. 2016) or null effects (Yang et al. 2014).

*P3* **.** The P3 is a maximal positive wave that typically peaks around 300 ms post-stimulus at the midline parietal region (Luck 2014). The P3 has attracted a lot of interest for its iconic increased positivity following the presentation of infrequent and surprising (low probability) stimuli (Pritchard 1981, Polich 2007). Although P2 and P3 are larger for infrequent stimuli, the P2 effect occurs only when the target is defined by simple stimuli, whereas the P3 effect can occur for complex stimuli (Song et al. 2005, Barkaszi et al. 2013, Luck 2014). Furthermore, ERP studies of self-referential processing show that P3 is often associated with the discrimination of self from others: a larger P3 wave follows the presentation of self-related objects, words, names, and faces relative to the same stimuli of other persons (Gray et al. 2004, Miyakoshi et al. 2007). Thus, the amplitude of the P3 might reflect increased attention or deeper processing of self-relevant stimuli (Porter et al. 2021).

### Hypotheses

We assessed behavioural (reaction times) and neurophysiological (P1, N170, P2, and P3) responses to positive and negative traits indicative of the authentic and presented selves in a modified Emotional Stroop Task. In terms of the presented self, we hypothesized an amplified emotional Stroop effect alongside an elevated P1 and N170 for negative versus positive traits. In terms of the authentic self, we offered competing hypotheses. First, according to the self-enhancement view, we would observe the same behavioural (amplified emotional Stroop effect) and neurophysiological (elevated P1 and N170) pattern as for the authentic self. However, according to the self-consistency/self-accuracy views, we would observe an attenuated emotional Stroop effect alongside an attenuated P1 and N170 for negative versus positive traits. We approached the ERPs for the P2 and P3 exploratorily, due to mixed findings regarding emotional and self-referential processing (P2) and lack of electrophysiological studies on authenticity (P3).

## Method

### Design and participants

We implemented a 3 (self: authentic self, presented self, control) × 2 (valence: positive traits, negative traits) within-subjects design. We used G*Power (Faul et al. 2009) assuming a small effect size (Cohen’s *f* = .10), six measures (based on the 3 × 2 design), α = .05, power (1−*β*) = .80, and a moderate correlation among the repeated measures (*r* = .50). Based on these parameters, a minimum *N *= 109 was required. We decided to recruit participants throughout the academic year, testing 162 University of [MASKED] undergraduate psychology student volunteers. Based on a-priori criteria, we excluded 15 participants: five encountered EEG acquisition device failures (Tacikowski and Nowicka 2010), two evinced over 50% missing data after cutting the 1% slowest and 1% fastest correct trials (Ratcliff 1993), two manifested mean reaction time exceeding ± 3 SDs (Cai et al. 2016), and six had more than 50% of their trials rejected due to artefacts in the EEG data (Imbir et al. 2021). The final sample comprised 147 participants (114 women, 31 men, 2 non-binary) aged 18–46 years (*M *= 19.56, SD = 2.87). We did not collect ethnicity information, but over 90% of the university’s undergraduate students are White. Sensitivity analyses (G*Power; Faul et al. 2009) indicated 80% power to detect effects as small as Cohen’s *f *= .086 (η^2^ = .007).

### Stimuli and procedure

To generate stimulus materials, we relied on Anderson’s (1968) list, a compendium of personality traits rated for likableness and meaningfulness. We selected 60 positive and 60 negative traits. The likableness of the selected positive traits (*M *= 4.66, SD = 0.45) was higher than the likableness of the selected negative traits (*M *= 1.25, SD = 0.46), *t*(118) = 40.89, *P *< .001, Cohen’s *d *= 7.47. The word length of the selected positive traits (*M *= 7.78, SD = 1.09) was not significantly different from the selected negative traits (*M *= 7.68, SD = 1.07), *t*(118) = 0.51, *P *= .612. We programmed and administered the experiment using PsychoPy (Version 2021.2.3; Peirce 2007).

Participants completed a modified emotional Stroop task in a quiet laboratory environment via computer and in the context of a larger study. (Participants also completed a Flanker task and a Monetary Incentive Delay Task. At the end, they completed a battery of personality questionnaires unrelated to the current investigation.) They were presented with a series of sentences and instructed to name the colour of each sentence, while ignoring its meaning, by pressing corresponding keys (V for red, B for blue, N for green) as quickly and accurately as possible. One third of these sentences described the authentic self (e.g. ‘I am genuinely ingenious’), one third described the presented self (e.g. ‘I am outwardly unkind’), and one third constituted the control condition (e.g. ‘It is clearly honest’) encompassing both positive and negative traits. We administered 360 trials across four blocks. Each block of 90 trials included an equal number of authentic self, presented self, and control trials. Each of these three trial sets consisted for an equal number of positive and negative traits. In all, there were 60 trials in each of the following bins: authentic self/positive, authentic self/negative, presented self/positive, presented self/negative, control/positive, control/negative. A trial started with the presentation of a central fixation cross for 800–1200 ms. Then, the colour sentence appeared on the screen until a response (key-pressing) occurred, followed by an 800 ms inter-stimulus interval (Fig. 1 for the trial event diagram). Prior to the formal task, participants underwent 12 practice trials to familiarize themselves with the colour-key mapping.

### Data recording and data analysis

We collected the EEG data from 32 scalp sites using Ag/AgCl electrodes embedded in a flexible cap (Brain Products, Germany), with an online reference to Cz. We mounted a ground electrode positioned at Fpz. We recorded the vertical electrooculogram (VEOG) below the right eye, based the electrode cap on the 10–20 system, and kept electrode impedances below 10 kΩ. We amplified and sampled the signals at 500 Hz with an online bandpass filter from DC to 140 Hz (−6 dB point, half-amplitude cutoff).

In offline processing, we initially pre-processed the EEG data by using EEGLAB, an open-source toolbox running in the MATLAB environment (Delorme and Makeig 2004). We digitally filtered the EEG data with a band-pass filter (high pass: 0.10 Hz, low pass: 40 Hz, 50 Hz notch; −6 dB point, half-amplitude cutoff) with a zero-phase FIR (finite impulse response) filter via Hamming-windowed sinc filtering. The filter order was automatically determined based on the transition bandwidth by the EEGLAB defaults. We segmented the EEG data from 200 ms prior to 800 ms following the onset of each word, and baseline corrected them to the 200 ms pre-stimulus baseline along with re-referencing them to the mastoids average (TP9, TP10). We detected bad channels by visual inspection of the waveforms and replaced them with a spherical spline identified interpolation (SSI; Perrin et al. 1989). We corrected segments contaminated by blinks, eye movements, and other artefacts using an independent component analysis (ICA) algorithm (e.g. fixed-point ICA, joint approximate diagonalization of eigen-matrices; Delorme and Makeig 2004) and applied ICLabel—a proposed statistical model—to automatically label ICs (Pion-Tonachini et al. 2019). Also, we excluded bad segments where a voltage deviation on any channel of ± 75 μV from the average baseline voltage.

Following best practices (Luck and Gaspelin 2017) and similar lines of research, we quantified: (a) P1 as the average peak amplitude from 80 to 130 ms after stimulus onset over lateral occipital electrode cluster (O1, OZ, O2; Wieser and Moscovitch 2015, Jetha et al. 2021); (b) N170 as the average peak amplitude from 140 to 200 ms after stimulus onset over lateral posterior electrode cluster (P3, P4, P7, P8; Keyes et al. 2010, Cai et al. 2016); (c) P2 as the average peak amplitude from 150 to 250 ms after stimulus onset over midline region (Fz, Cz, Pz; Fan et al. 2013, 2016); and (d) P3 as the average peak amplitude from 300 to 400 ms after stimulus onset over midline region (Fz, Cz, Pz; Gray et al. 2004, Wada et al. 2019, Riggins and Scott 2020).

The main dependent variables were reaction times (RT) and ERPs (P1, N170, P2, P3). We only used correct responses (Montalan et al. 2008). We created a RT data processing pipeline (Morís Fernández and Vadillo 2020). Specifically, we removed: (a) the 1% slowest and 1% fastest trials; (b) participants with more than 50% missing data; (c) participants with a mean RT exceeding ± 3 SDs. Further, we averaged the ERPs for each of the six experimental conditions. We analyzed the data in SPSS (Version 24), addressing multiple comparisons with Bonferroni corrections.

## Results

We conducted 3 (self) × 2 (valence) repeated measures Analyses of Variance on RT and ERPs (P1, N170, P2, P3).

### Reaction times

The main effect of self was significant, *F*(2, 145) = 4.40, *P* = .014, *ƞ*_p_^2^ = .06. Participants responded faster on authentic-self (*M *= 625.05, SD = 108.79) than presented-self (*M *= 631.28, SD = 109.59) traits, *P* = .028, 95% CI = [−11.97, −0.49]. They did not differ in their speed of responding to control (*M *= 626.72, SD =106.26) and presented-self (*M *= 631.28, SD = 109.59) traits, *P* = .071, 95% CI = [−0.27, 9.38], or control and authentic-self traits, *P* = .999, 95% CI = [−7.60, 4.25]. Further, the main effect of valence was significant. As per the emotional Stroop effect (Williams et al. 1996), participants responded slower to negative (*M *= 629.97, SD = 109.54) than positive (*M *= 625.39, SD = 106.22) traits, *F*(1, 146) = 4.19, *P* = .042, *ƞ*_p_^2^ = .03.

**Figure 2.**
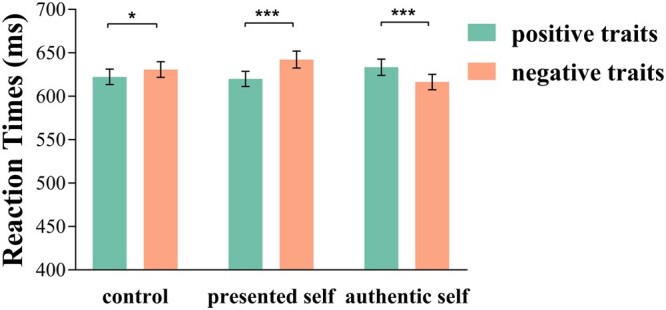
Reaction times to positive traits and negative traits for control, presented self, and authentic self.

Crucially, the Self × Valence interaction was significant, *F*(2, 145) = 30.88, *P* < .001, *ƞ*_p_^2^ = .30 (Fig. 2). The prototypical emotional Stroop effect emerged on control trials: Participants were slower to respond to negative (*M *= 630.96, SD = 109.53) than positive (*M *= 622.48, SD = 107.13) traits, *P* = .016, 95% CI = [1.60, 15.37]. The emotional Stroop effect was amplified on presented-self trials: Participants were even slower to respond to negative (*M *= 642.40, SD = 117.98) than positive (*M *= 620.16, SD = 105.17) traits, *P* < .001, 95% CI = [15.10, 29.38]. Finally, the emotional Stroop effect was attenuated on authentic-self trials: Participants responded faster to negative (*M *= 616.55, SD = 108.29) than positive (*M *= 633.54, SD = 112.77) traits, *P* < .001, 95% CI = [−23.41, −10.58].

### Event-related potentials

We observed a pronounced emotional Stroop effect for the presented self and a diminished emotional Stroop effect for the authentic self. We next turned to ERPs relevant to early-stage selective attention (P1), attention allocation to emotionally evocative stimuli (N170), and early (P1) and late (P3) stages of self-relevant information processing in search of an explanation for this behavioural effect (the Self × Valence interaction on RTs).

**Figure 3.**
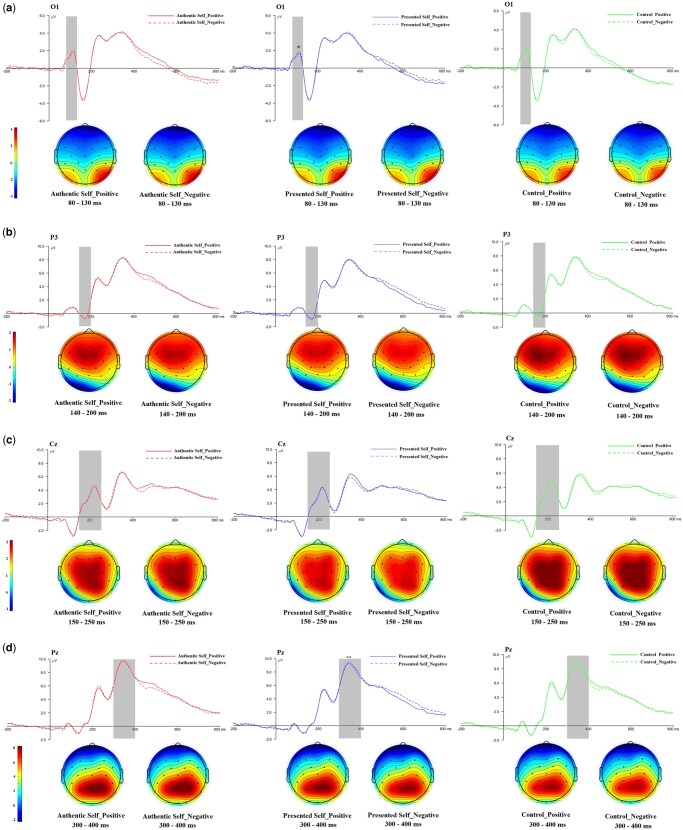
Grand averages waveforms and topographies of ERPs (P1: a; N170: b; P2: c; P3: d) in the authentic self, presented self, and control conditions.

***P1*.** The Self × Valence interaction was significant, *F*(2, 145) = 3.75, *P* = .026, *ƞ*_p_^2^ = .05 (Fig. 3a). Negative traits (*M *= 4.19, SD = 3.77) elicited a larger P1 than positive traits (*M *= 3.91, SD = 3.71) for the presented self, *P* = .019, 95% CI = [0.05, 0.53]. The P1 did not differ between negative (*M *= 3.97, SD = 3.92) and positive (*M *= 3.98, SD = 3.74) traits for the authentic self, *P *= .919, 95% CI = [−0.27, 0.30], nor did it differ between negative (*M *= 3.67, SD = 3.67) and positive (*M *= 3.98, SD = 3.68) control traits, *P* = .052, 95% CI = [0.00, 0.62]. Neither the main effect of self, *F*(2, 145) = 1.50, *P* = .227, *ƞ*_p_^2^ = .02, nor that of valence, *F*(2, 145) = 0.03, *P* = .855, *ƞ*_p_^2^ < .001, was significant.

***N170*.** The main effect of self was significant, *F*(2, 145) = 3.71, *P* = .027, *ƞ*_p_^2^ = .05 (Fig. 3b). The N170 was larger for the presented self (*M *= −2.31, SD = 2.57) than control (*M *= −2.11, SD = 2.73) traits, *P* = .027, 95% CI = [−0.39, −0.02]. Presented-self and authentic-self (*M *=* −*2.28, SD = 2.56) traits did not differ, *P* = .999, 95% CI = [−0.19, 0.14] and neither did authentic-self and control traits, *P* = .088, 95% CI = [−0.37, 0.02]. The main effect of valence, *F*(1, 146) = 1.80, *P* = .182, *ƞ*_p_^2^ = .01, and the Self × Valence interaction, *F*(2, 145) = 0.37, *P* = .695, *ƞ*_p_^2^ = .01, were not significant.

***P2.*** The main effect of self was significant, *F*(2, 145) = 27.27, *P* < .001, *ƞ*_p_^2^ = .27 (Fig. 3c). The P2 was larger on control traits (*M *= 6.58, SD = 3.53) compared to both authentic-self (*M *= 6.23, SD = 3.42) and presented-self (*M *= 5.93, SD = 3.47) traits, *P* = .004, 95% CI = [0.09, 0.61] and *P* < .001, 95% CI = [0.43, 0.86]. These results are consistent with findings showing substantially reduced P2 amplitudes for self-relevant stimuli (Keyes et al. 2010, Liu et al. 2019). The P2 was also larger on authentic-self compared to presented-self traits, *P* = .008, 95% CI = [0.06, 0.53]. Thus, we observed a linear pattern where the P1 was largest for control traits, intermediate for authentic-self traits, and smallest for presented-self traits. Neither the main effect of valence, *F*(1, 146) = 0.05, *P* = .993, *ƞ*_p_^2^ < .001, nor the Self × Valence interaction, *F*(2, 145) = 0.06, *P* = .940, *ƞ*_p_^2^ = .001, was significant.

***P3.*** The main effect of self was significant, *F*(2, 145) = 16.07, *P* < .001, *ƞ*_p_^2^ = .18. (For P3, the results of three electrodes separately differed somewhat from the analysis that averaged all three electrode sites together. Specifically, we obtained the Self × Valence interaction at FZ and CZ, but not at PZ. For detailed results of each electrode, see the Supplementary Materials (pp. 2–3).) The P3 was larger on authentic-self (*M *= 7.54, SD = 4.12) than presented-self (*M *= 7.20, SD = 4.28) traits, *P* = .026, 95% CI = [0.03, 0.66] and control (*M *= 6.87, SD = 4.20), *P* < .001, 95% CI = [0.39, 0.97] traits. The P3 was larger on presented-self compared to control traits, *P* = .021, 95% CI = [0.04, 0.063]. The finding that P3 was larger for self-relevant stimuli is compatible with the literature (Gray et al. 2004, Miyakoshi et al. 2007, Tacikowski and Nowicka 2010). The main effect of valence was not significant, *F*(1, 146) = 1.62, *P* = .205, *ƞ*_p_^2^ = .01. However, the important Self × Valence interaction was significant, *F*(2, 145) = 4.07, *P* = .019, *ƞ*_p_^2^ = .05 (Fig. 3d). The P3 did not differ between negative and positive traits in the control (*P* = .346, 95% CI = [−0.19, 0.54]) and authentic-self (*P* = .187, 95% CI = [−0.56, 0.11]) condition. Yet, the P3 was larger for positive (*M *= 7.41, SD = 4.37) than negative (*M *= 6.99, SD = 4.18) traits in the presented-self condition, *P* = .007, 95% CI = [0.12, 0.73]. (Although the Self × Valence hypothesis posits a stronger P3 to negative words due to their lower expectedness or congruence (Pritchard 1981, Polich 2007), the evidence more robustly aligns with the self-enhancement view. First, the main effect of self revealed a larger P3 for both self conditions compared to control. This effect likely reflects enhanced attention and processing of self-relevant information relative to control rather than increased surprise or unexpectedness for self-related stimuli. Second, the absence of a significant main effect of valence contradicts the unexpectedness account, which would predict larger P3 responses to negative words due to their presumed incongruence. Finally, simple effects analyses of the Self × Valence interaction showed that for positive words, the P3 was larger for both the authentic self and the presented self compared to the control condition, whereas for negative words, the P3 was larger for the authentic self than for both the presented self and the control condition. Interpreted through the lens of unexpectedness, this would imply that positive and negative words in self-relevant conditions (authentic self and presented self) were more surprising than in the non-self-relevant condition (control) condition), a counterintuitive proposition.) Alternatively, when processing positive words, the P3 was larger for both the authentic (*M *= 7.43, SD = 4.02; *P *= .004, 95% CI = [0.12, 0.83]) and presented (*M *= 7.41, SD = 4.37; *P *= .047, 95% CI = [0.004, 0.91]) self relative to the control condition (*M *= 6.95, SD = 4.13), with the authentic- and presented-self conditions not differing significantly, *P *= .999, 95% CI = [−0.41, 0.45]. In contrast, when processing negative words, the P3 was larger for the authentic-self (*M *= 7.66, SD = 4.23) than for both the presented-self (*M *= 6.99, SD = 4.18; *P *< .001, 95% CI = [0.26, 1.08]) and control (*M *= 6.78, SD = 4.27; *P *< .001, 95% CI = [0.42, 1.33]) condition, with the authentic- and presented-self conditions not differing significantly, *P *= .650, 95% CI = [−0.20, 0.61].

## Discussion

We decomposed the self into two mental representations, presented and authentic. Accordingly, we examined competing views of authenticity (self-enhancement vs. self-consistency/self-accuracy) using behavioural and neurophysiological measures in a modified Emotional Stroop Task. Results largely favoured the self-consistency/self-accuracy views. On control trials, we demonstrated a prototypical emotional Stroop effect (slowdown for negative compared to positive information). This effect was amplified on presented-self trials, attributable to the potent self-enhancement motivation driving self-presentation (Schlenker and Pontari 2000, Alicke and Sedikides 2009, Sedikides et al. 2015). On authentic-self trials, however, the emotional Stroop effect was attenuated, as per the self-consistency/self-accuracy views.

The ERP results help to explain the behavioural effects. The earliest stages of selective attention (P1) largely echoed our behavioural findings. Negative (vs. positive) traits elicited a larger P1 for the presented self. However, there was no difference in P1 amplitudes between negative and positive traits for the authentic self, a pattern compatible with the self-consistency/self-accuracy views. The P1 is sensitive to emotional stimuli (Mueller et al. 2013, Schindler and Bublatzky 2020), especially threat-related information, representing an evolutionary adaptation for threat vigilance (Luo et al. 2010, Zhang et al. 2014). Therefore, these findings indicate that the presented self is strongly motivated by self-enhancement and is consequently more susceptible to threatening information. Accordingly, attentional resources are involuntarily allocated towards negative self-descriptive stimuli during the early stages of attentional allocation. In contrast, the authentic self acknowledges both strengths and weaknesses (Kernis and Goldman 2006), rendering it possible to distribute attentional resources more evenly between positive and negative traits. Moreover, the literature indicates that emotional stimuli are rapidly processed in the early attention stage, with self-referent processing arising later (Herbert et al. 2011, Zhu et al. 2016, Schäfer et al. 2020). Hence, our P1 findings may represent an initial demonstration of self-relevant modulation of emotional processing in this early attention stage. This modulation, if replicated by future work, would mark a novel addition to understanding of the interplay between the self and emotional processing in this early attention stage. Although some psycholinguistic research (Sereno et al. 2003) suggests minimal influence of lexical-semantic properties on early ERP components, we observed significant P1 modulation, which may be attributed to the Emotional Stroop task we employed. Specifically, by restricting task demands to low-level perceptual processing (colour identification) while presenting emotionally charged words, we ensured that emotional processing remained implicit—an automatic, unconscious processing that may enhance the ability to detect early-stage automatic attentional biases towards emotional stimuli. Researchers could conduct independent replications of the P1 effect to verify its robustness.

Although the N170 component did not differentiate between positive and negative self-descriptiveness, N170 amplitudes were larger for presented self than control trials, a pattern that warrants further exploration. Overall, these findings suggest that the presented self selectively heightens attention to negativity during early processing, whereas the authentic self lowers selective attention to negativity. We carefully matched stimuli across emotion conditions for basic lexica properties known to influence early ERPs (Hauk and Pulvermüller 2004). For example, the word length of the selected positive and negative traits was not significantly different. Moreover, the implementation of within-subjects design addresses potential issues arising from orthographic neighbourhood and bigram frequency.

We observed an interaction between self and valence at the later processing stage, the P3 (but not P2). Whereas there was no difference between the P3 to negative versus positive traits for the authentic self (as per the self-consistency/self-accuracy views), the P3 was larger for positive than negative traits for the presented self (as per the self-enhancement view). Also, although both P2 and P3 are larger for infrequent and salient stimuli, modulation of the P2 occurs only when the target is defined by simple stimuli, but modulation of the P3 can occur for complex stimuli (Song et al. 2005, Barkaszi et al. 2013, Luck 2014), as stated earlier. The presence of the Self × Valence interaction for the P3 (and not the P2) bolsters the representational richness of the self (Kihlstrom et al. 1988, Sedikides and Gregg 2003, McConnell 2011). Moreover, the lack of difference in the P3 response to negative and positive traits for the authentic self suggests that, at this later processing stage, negativity and positivity are comparably relevant to the authentic self. Similarly, the larger P3 for positive (vs. negative) traits for the presented self indicates that, at this stage, positivity is novel and salient to the presented self. This shift contrasts with the earlier stage (P1), where the presented self exhibited heightened sensitivity to negativity. This pattern can be accounted for by the mobilization-minimization hypothesis (Taylor 1991; see also Sedikides et al. 2016), according to which negative or threatening information triggers swift physiological, cognitive, emotional, and social responses (i.e. mobilization), followed by counteractions to minimize, undo, or even reverse these initial responses (i.e. minimization). In our research, negative self-descriptive information received preferential processing initially (mobilization; P1), followed by preferential processing of positive, self-descriptive information (minimization; P3).

We observed the reverse P3 pattern in the frontal-central rather than parietal region, in line with neuroimaging evidence that self-referential processing predominantly activates cortical midline structures (CMS; Northoff and Bermpohl 2004, Uddin et al. 2007). Specifically, self-referential emotional processing involves anterior CMS regions such as the ventromedial prefrontal cortex, anterior cingulate cortex, and dorsomedial prefrontal cortex (Northoff et al. 2006). Thus, the frontal-central distribution of our reverse P3 effects in the Emotional Stroop Task with self-referential stimuli provides electrophysiological support for this CMS framework.

The main effects of self enrich understanding of the P3. ERP studies of self-referential processing show that the P3 is frequently larger following presentation of self-relevant objects, words, names, and faces relative to identical stimuli describing another person (Gray et al. 2004, Miyakoshi et al. 2007, Tacikowski and Nowicka 2010). Consequently, the P3’s amplitude might reflect increased attention or deeper processing of self-relevant stimuli (Porter et al. 2021). We replicated this finding by demonstrating that P3 was larger for the authentic and presented self than in the control trials. We then extended this finding by illustrating that the effects of self-reference on the P3 are stronger for the authentic self. Hence, deeper processing of self-relevant stimuli (Porter et al. 2021, Levorsen et al. 2023) may be driven more by the authentic than presented self. Future investigations might address this possibility.

## Limitations

We did not provide participants with operational definitions of the terms ‘genuinely’ and ‘outwardly’, instead relying on their lexical comprehension. Although these terms lexically map onto the constructs of ‘authentic self’ and ‘presented self’ (‘Genuine’ denotes authenticity: ‘Having the character or origin represented; real, true, not counterfeit, unfeigned, unadulterated’ (Oxford University Press, 2025a). ‘Outward’ denotes the presented self: ‘Of or relating to thoughts, attitudes, actions, etc, which manifest externally, as opposed to those which are experienced internally; merely exterior; public. Also: of or relating to the outer or visible form of something’ (Oxford University Press, 2025b).), respectively, this approach may have introduced variability in how participants interpreted them. To increase methodological precision, future research could clarify such manipulations by defining the terms and ensuring their comprehension via pretesting.

Also, we used whole-sentence rather than word-by-word presentation. This approach may increase ocular artefacts compared to word-by-word presentation and introduce variability in the precise timing of word processing. Given EEG’s temporal precision (Cohen 2017), even a 100 ms difference in when participants read the critical stimuli could impact both the selection of analysis time windows and the interpretation of observed components as reflecting specific neural processes. However, alternative sequential presentation of self-referential and emotional words would create its own confounds: congruent colours between words might produce facilitation effects, whereas incongruent colours could lead to interference in colour judgement. Future studies might implement more sophisticated designs, such as rapid serial visual presentation or masked priming approaches, to simultaneously address both temporal precision and artefact minimization concerns.

## Conclusion

Distinct self-representations, the authentic and presented selves, are differential susceptibility to negative self-relevant information, behaviourally and neurophysiologically. From the self-enhancement view, the presented self is particularly vulnerable to negative self-descriptors. In contrast, from the self-consistency/self-accuracy views, the authentic self integrates both positive and negative self-aspects with comparable weight. The findings aligned with the latter views, suggesting that authenticity extends beyond mere self-enhancement.

## Supplementary Material

nsaf103_Supplementary_Data

## Data Availability

All data, code, and materials used in the analysis are available on Open Science Framework (https://osf.io/64sq8/? view_only=99c43f1d13774d30ace7879a5cecbfcc).

## Supplementary Material

See the online supplementary material for detailed stimulus validation procedures, additional analyses, and figures.
