# Supplementary material for: Authenticity is more than self-enhancement: behavioural and neurophysiological evidence
Source: Soc Cogn Affect Neurosci. 2025 Oct 14;20(1):nsaf103. doi: 10.1093/scan/nsaf103 (PMC12687596; doi:10.1093/scan/nsaf103)
Supplement: nsaf103_Supplementary_Data [file nsaf103_supplementary_data.zip › Supplementary Materials.docx]

**Authenticity is More Than Self-Enhancement:**

**Behavioral and Neurophysiological Evidence**

Chengli Huang^1,2^, Emily K. Penney^2^, Constantine Sedikides^2^, and Nicholas J. Kelley^2^

^1^School of Mental Health, Wenzhou Medical University, China

^2^Centre for Research on Self and Identity, University of Southampton, UK

**Analyses of P3**

For Fz, the main effect of self was significant, *F*(2, 145) = 8.11, *p* < .001, *ƞ*_p_^2^ = .10. The P3 was larger on authentic-self than control traits (3.92 ± 4.72 vs 3.38 ± 4.85; *p* < .001, 95% *CI* = [0.22, 0.87]). We found no significant differences between presented-self and control traits (3.60 ± 4.86 vs 3.38 ± 4.85; *p* = .021, 95% *CI* = [0.04, 0.063]), or between authentic-self and presented-self traits (3.92 ± 4.72 vs 3.60 ± 4.86; *p* = .021, 95% *CI* = [0.04, 0.063]). The main effect of valence was not significant, *F*(1, 146) = 1.19, *p* = .277, *ƞ*_p_^2^ = .008. The Self × Valence interaction was significant, *F*(2, 145) = 5.14, *p* = .007, *ƞ*_p_^2^ = .07. The P3 did not differ between negative and positive traits in the control condition (*p* = .652, 95% *CI* = [-0.31, 0.50]) or in the case of the authentic self (*p* = .164, 95% *CI* = [-0.75, 0.13]). However, the P3 was larger for positive (3.90 ± 4.83) than negative (3.29 ± 4.88) traits in the case of the presented self, *p* = .002, 95% *CI* = [0.24, 1.00].

For Cz, the main effect of self was significant, *F*(2, 145) = 11.56, *p* < .001, *ƞ*_p_^2^ = .14. The P3 was larger on authentic-self than presented-self (7.86 ± 4.72 vs 7.50 ± 4.82; *p* = .045, 95% *CI* = [0.01, 0.73]) or control (7.86 ± 4.72 vs 7.15 ± 4.72; *p* < .001, 95% *CI* = [0.35, 1.67]) traits. The P3 was largest for presented-self than control traits (7.50 ± 4.82 vs 7.15 ± 4.72; *p* = .049, 95% *CI* = [0.001, 0.68]). The main effect of valence was not significant, *F*(1, 146) = 2.66, *p* = .105, *ƞ*_p_^2^ = .02. The Self × Valence interaction was significant, *F*(2, 145) = 4.21, *p* = .017, *ƞ*_p_^2^ = .06. The P3 did not differ between negative and positive traits in the control condition (*p* = .185, 95% *CI* = [-0.14, 0.72]) or in the case of the authentic self (*p* = .227, 95% *CI* = [-0.64, 0.15]). However, the P3 was larger for positive (7.74 ± 4.91) than negative (7.25 ± 4.72) traits in the case of the presented self, *p* = .007, 95% *CI* = [0.14, 0.86].

For Pz, the main effect of self was significant, *F*(2, 145) = 18.61, *p* < .001, *ƞ*_p_^2^ = .20. The P3 was larger on authentic-self than presented-self (10.84 ± 4.99 vs 10.50 ± 5.02; *p* = .020, 95% *CI* = [0.04, 0.64]) or control (10.84 ± 4.99 vs 10.07 ± 5.05; *p* < .001, 95% *CI* = [0.47, 1.08]) traits. The P3 was largest for presented-self than control traits (7.50 ± 4.82 vs 7.15 ± 4.72; *p* = .003, 95% *CI* = [0.12, 0.75]). Neither the main effect of valence, *F*(1, 146) = 0.34, *p* = .564, *ƞ*_p_^2^ = .002, nor the Self × Valence interaction, *F*(2, 145) = 0.90, *p* = .407, *ƞ*_p_^2^ = .01, was significant.

**Analyses of N400**

We quantified N400 as the average peak amplitude from 400–500 ms after stimulus onset at centro-parietal sites (i.e., Cz, Pz; Kutas & Federmeier, 2011; Luck, 2014).

The main effect of self was significant, *F*(2, 145) = 15.12, *p* < .001, *ƞ*_p_^2^ = .18. It was larger for control traits (*M* = 3.07, *SD* = 4.21) than both authentic-self (*M* = 3.81, *SD* = 3.92), *p* < .001, 95% *CI* = [0.42, 1.07] and presented-self (*M* = 3.60, *SD* = 4.12), *p* < .001, 95% *CI* = [0.20, 0.86] traits. There was no significant difference between authentic-self (*M* = 3.81, *SD* = 3.92) and the presented-self (*M* = 3.60, *SD* = 4.12) traits, *p* = .304, 95% *CI* = [-0.52, 0.10]. The main effect of valence was significant, *F*(1, 146) = 12.11, *p* = .001, *ƞ*_p_^2^ = .08. The N400 was larger (more negative) for negative traits (*M* = 3.31, *SD* = 4.02) compared to positive (*M* = 3.67, *SD* = 4.15) traits. The Self × Valence interaction was not significant, *F*(2, 145) = 1.96, *p* = .145, *ƞ*_p_^2^ = .03.

**References**

Kutas, M., & Federmeier, K. D. (2011). Thirty years and counting: finding meaning in the N400 component of the event-related brain potential (ERP). *Annual Review of Psychology*, *62*(1), 621–647. <https://doi.org/10.1146/annurev.psych.093008.131123>

Luck, S. J. (2014). *An introduction to the event-related potential technique* (2nd ed). MIT Press.
